# Protein disulfide isomerase family 6 promotes the imatinib-resistance of renal cell carcinoma by regulation of Wnt3a-Frizzled1 axis

**DOI:** 10.1080/21655979.2021.2005218

**Published:** 2021-12-07

**Authors:** Yong Huang, Ping He, Juan Ding

**Affiliations:** Department of Pharmacy, Hospital of Chengdu University of Traditional Chinese Medicine, Chengdu, Sichuan Province, China

**Keywords:** PDIA6, imatinib-resistant, renal cell carcinoma cells, Wnt3a-Fzd1

## Abstract

Imatinib is a nontoxic tyrosine kinase inhibitor, used in the treatment of advanced renal cell carcinoma. However, some patients with renal cell carcinoma develop resistance to imatinib. Protein disulfide isomerase family 6 (PDIA6) was involved in the chemo-resistance of lung adenocarcinoma. In this study, the effect of PDIA6 on imatinib-resistance of renal cell carcinoma was investigated. First, PDIA6 was found to be up-regulated in the imatinib-resistant renal cell carcinoma tissues and cells. Functional assays showed that knockdown of PDIA6 sensitized imatinib-resistant renal cell carcinoma cells to imatinib through decreasing the half-maximal inhibitory concentration (IC50) of imatinib-resistant renal cell carcinoma cells. Secondly, cell proliferation of imatinib-resistant renal cell carcinoma cells was suppressed by PDIA6 silencing, and the apoptosis was promoted with reduced Bcl-2, enhanced Bax and cleaved caspase-3. Moreover, the interference of PDIA6 increased phosphorylation of H2A histone family member X (γH2AX), while decreased Rad51 and phosphorylated DNA-dependent protein kinase (DNA-PK) (p-DNA-PK) in imatinib-resistant renal cell carcinoma cells. Lastly, protein expression levels of Wnt3a and Frizzled1 (FZD1) in imatinib-resistant renal cell carcinoma cells were down-regulated by silencing of PDIA6. Over-expression of FZD1 attenuated PDIA6 silencing-induced increase in cell apoptosis and decrease in cell proliferation in imatinib-resistant renal cell carcinoma cells. In conclusion, knockdown of PDIA6 sensitized imatinib-resistant renal cell carcinoma cells into imatinib through inactivation of Wnt3a-FZD1 axis.

## Introduction

Renal cell carcinoma is the most common type of kidney cancer, accounting for approximately 85% of all kidney cancers [1]. The incidence and mortality of renal cell carcinoma have been increasing in recent years [2]. Renal cell carcinoma is a highly metastatic urinary tumor and originates from the renal cortex. Surgery, immunotherapy, or targeted therapies can improve the 5-year overall survival of renal cell carcinoma to 70%–80% [3]. However, the metastatic or advanced renal cell carcinoma is resistant to the targeted therapies [4]. Novel therapeutic strategies are urgently needed to ameliorate drug resistance in renal cell carcinoma.

Imatinib, which is an inhibitor of BCR-ABL, platelet-derived growth factor receptor, and c-kit tyrosine kinase, is a molecular targeted therapy drug approved for the treatment of advanced gastrointestinal stromal tumors [5]. Imatinib has also been used in the treatment of renal cell carcinoma, and the drug resistance reduced the treatment efficiency [6,7].

Protein disulfide isomerase family 6 (PDIA6) is a member of the protein disulfide bond isomerase family, which is primarily located in the endoplasmic reticulum, and functions as an oxidoreductase to catalyze the formation of disulfide bonds and serves as a chaperone to assist protein folding and inhibit the aggregation of unfolded substrates [8]. Recent studies have reported that PDIA6 is implicated in the pathogenesis of human cancers. For example, PDIA6 was upregulated in non-small cell lung cancer cells and inhibited apoptosis and autophagy of cancer cells [9]. PDIA6 also promoted the proliferation and migration of glioma cells [10], and contributed to the aerobic glycolysis of oral squamous cell carcinoma [11]. Down-regulation of PDIA6 suppressed cell proliferation and invasion of bladder cancer [12]. However, the effect of PDIA6 on progression of renal cell carcinoma has not been reported yet. In addition, PDIA6 has also been shown to be a chemical-resistant factor. PDIA6 increased the resistance of lung adenocarcinoma cells to chemotherapeutic drugs through inhibition of apoptosis and autophagy of cancer cells [13], and inhibition of PDIA6 promoted the sensitivity of gastric cancer cells to cisplatin [14]. In this study, we hypothesized that PDIA6 promoted the imatinib resistance of renal cell carcinoma. The effects of PDIA6 on proliferation, apoptosis, and DNA damage repair of imatinib-resistance of renal cell carcinoma were investigated. The results might provide a novel strategy for chemotherapeutic resistant renal cell carcinoma.

## Materials and methods

### Tumor tissues

A total of 60 patients with renal cell carcinoma (including 24 patients with imatinib-resistance and 36 with imatinib-sensitivity) were recruited to the Hospital of Chengdu University of Traditional Chinese Medicine. Patients without recurrence for more than 6 months after imatinib therapy were regarded as imatinib sensitive, while patients with recurrence less than 6 months after the therapy were regarded as imatinib resistance. The clinical information of these 60 patients are shown in Table 1. The renal cell carcinoma tissues and the normal tissues were collected from the patients with written informed consents from 2016 to 2019 by surgical excision. The study was approved by the Ethics Committee of the Hospital of Chengdu University of Traditional Chinese Medicine (Approval no. 2016121) and in accordance with the World Medical Association Declaration of Helsinki. Ethical principles for medical research involving human subjects.

**Table 1.** Clinical information of the 60 patients included in this study

Characteristics	Patients with imatinib sensitivity		Patients with imatinib resistance	
	Number (%)		Number (%)	
Age 55 (25–72) years <35 35–55 >55 Tumor Size (cm) ≤4 >4 T stage T1-2 T3-4 Fuhrman I,II III,IV Metastasis Negative Positive	36 10 21 5 11 25 22 14 19 17 20 16	27.8 58.3 13.9 30.6 69.4 61.1 38.7 52.8 47.2 55.6 44.4	24 2 4 18 10 14 8 16 10 14 9 15	8.3 16.7 75.0 41.7 58.3 33.3 66.7 41.7 58.3 37.5 62.5

### Cell culture, treatment, and transfection

Renal cell carcinoma cell line, A498, was purchased from American Type Culture Collection (Manassas, VA, USA). Cells were cultured in Dulbecco’s modified Eagle’s medium containing 10% fetal bovine serum and penicillin–streptomycin (Gibco-BRL, Carlsbad, CA, USA) in 37°C incubator. For the establishment of imatinib-resistant A498 (A498/R), A498 cells were incubated with 1 mM imatinib (Sigma-Aldrich, Shanghai, China), and the concentration of imatinib was increased by 1 mM per week. The cells were treated with an increased concentration of imatinib for 5 months. Five months later, A498 cells were incubated with 20 mM imatinib for another 1 month to establish A498/R cell line. A498/R was transfected with shRNA (short hairpin RNA) targeting PDIA6 (shPDIA6) or the negative control (shNC) (Genepharma, Shanghai, China) by Lipofectamine 2000 (Invitrogen, Carlsbad, CA, USA). A498/R was also cotransfected with shPDIA6 and pcDNA-FZD1 by Lipofectamine 2000. The transfection efficiency was confirmed by western blot. Two days later, the cells were treated with 10 mM imatinib for 24 hours before functional assays [15].

### Cell viability

A498 or A498/R were seeded in a 96-well plate containing 0.5, 1, 2, 4, 8, 16, 32, 64, 128, or 256 μM imatinib for 24 hours. Cell Counting Kit 8 (CCK8) solution (Dojindo, Tokyo, Japan) was added into each well and incubated for 2 hours [15]. Absorbance at 450 nm was measured by Thermo Multiskan MK3 (Thermo Fisher Scientific Inc, Waltham, MA, USA).

### Cell proliferation and apoptosis assays

A498/R cells with indicated transfections were seeded in a 6-well plate and treated with or without 10 mM imatinib. Ten days later, the cells were fixed in methanol and stained with crystal violet before photographed under a light microscope (Olympus, Tokyo, Japan). For cell apoptosis analysis, A498/R cells were harvested, and then suspended in a binding buffer from ApoDETECT Annexin V-FITC Kit (Thermo Fisher Scientific Inc.). The cells were incubated with Annexin V-FITC and PI (Thermo Fisher Scientific Inc) and then the cell apoptosis was analyzed by FACS flow cytometer (Attune, Life Technologies, Darmstadt, Germany) [16].

### Immunofluorescence

A498/R cells were fixed in 4% paraformaldehyde, and then permeabilized in 0.2% Triton/PBS. The cells were blocked with 5% BSA, and then incubated with anti-γH2AX monoclonal antibody (Abcam, Cambridge, MA, USA). Following incubation with Alexa488-conjugated goat anti-human secondary antibody (Abcam), the cells were photographed under a fluorescence microscope (Nikon, Tokyo, Japan) with 4,6-diamidino-2-phenylindole staining [17].

### Quantitative Reverse Transcription PCR (qRT-PCR)

RNAs were isolated from renal cell carcinoma tissues (100 mg) and cells (1 × 10^6^) via Trizol (Invitrogen). The RNAs were reverse-transcribed into cDNAs and conducted with qRT-PCR analysis of PDIA6 using SYBR Green Master (Roche, Mannheim, Germany) [15]. The following primers for PDIA6 (forward: 5ʹ-tttctgataacgccccacct-3ʹ; reverse: 5ʹ-ccaaggctctctctcaacga-3ʹ) and GAPDH (forward: 5ʹ-tgaccacagtggatgccat-3ʹ; reverse: 5ʹ-ttactccttggaggccatgt-3ʹ), were used in this study. GAPDH was used as endogenous control.

### Western blot

Proteins extracted from the renal cell carcinoma tissues and cells were separated by sodium dodecyl sulfate-polyacrylamide gel electrophoresis and transferred onto polyvinylidene difluoride membrane. The membranes were blocked and probed with primary antibodies, including anti-PDIA6 and anti-GAPDH (1:2000; Abcam, Cambridge, MA, USA), anti-Bax and anti-Bcl-2 (1:2500; Abcam), anti-Bax and anti-Bcl-2 (1:3000; Abcam), anti-cleaved caspase-3 and anti-p-DNA-PK (1:3500; Abcam), as well as anti-Rad51 (1:4000; Abcam). Following incubation with the corresponding secondary antibodies (1:5000; Abcam), the signals of protein bands were measured by Colorimetric Western Blotting Kit (Sigma-Aldrich) [15].

### Statistical analysis

All the data from at least triplicate experiments were expressed as mean ± S.D. and analyzed by student’s *t* test or one-way analysis of variance. A *p* value of less than 0.05 was considered as statistically significant.

## Results

To investigate the role of PDIA6 in imatinib resistance of renal cell carcinoma, the expression level of PDIA6 in imatinib-resistant or imatinib-sensitive renal cell carcinoma tissues and cells was first detected. The results showed that PDIA6 was up-regulated in imatinib-resistant renal cell carcinoma. Knockdown of PDIA6 suppressed cell proliferation and DNA damage repair and promoted the apoptosis of imatinib-resistance of renal cell carcinoma through inactivation of activation of Wnt3a-FZD1 signaling.

### PDIA6 was up-regulated in imatinib-resistant renal cell carcinoma

**Figure 1.**
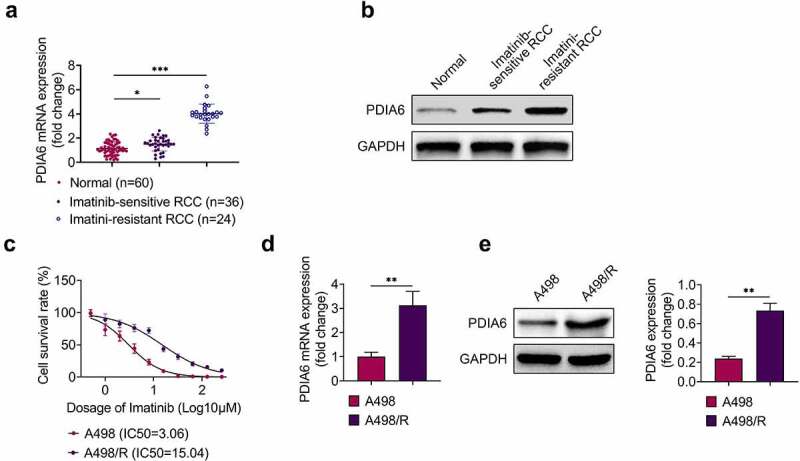
PDIA6 was up-regulated in imatinib-resistant renal cell carcinoma (a) PDIA6 mRNA was increased in renal cell carcinoma tissues compared with the normal tissues, and enhanced in imatinib-resistant renal cell carcinoma tissues compared to the imatinib-sensitive renal cell carcinoma tissues. (b) PDIA6 protein was increased in renal cell carcinoma tissues compared to the normal tissues and enhanced in imatinib-resistant renal cell carcinoma tissues compared to the imatinib-sensitive renal cell carcinoma tissues. (c) IC50 was significantly increased in A498/R (15.04 μM) compared to A498 (3.06 μM). (d) PDIA6 mRNA was upregulated in A498/R cells compared to A498. (e) PDIA6 protein was upregulated in A498/R cells compared to A498. **, ***, *p* < 0.01, *p* < 0.001

Expression of PDIA6 in renal cell carcinoma was detected to investigate the role of PDIA6 in renal cell carcinoma. Data from qRT-PCR (Figure 1(a)) and western blot (Figure 1(b)) showed that PDIA6 was increased in renal cell carcinoma tissues compared with the normal tissues. Moreover, PDIA6 was also enhanced in imatinib-resistant renal cell carcinoma tissues (n = 24) compared with the imatinib-sensitive renal cell carcinoma tissues (n = 36) (Figure 1(a,b)). Renal cell carcinoma cells (A498) were incubated with increasing concentration of imatinib to establish imatinib-resistant A498 (A498/R). IC50 was significantly increased in A498/R (15.04 μM) compared to A498 (3.06 μM) (Figure 1(c)), confirming the resistance of A498/R to imatinib. Additionally, PDIA6 was also up-regulated in A498/R cells (Figure 1(d,e)), suggesting that PDIA6 might be involved in imatinib-resistance of renal cell carcinoma.

### Knockdown of PDIA6 suppressed cell proliferation of imatinib-resistant renal cell carcinoma

**Figure 2.**
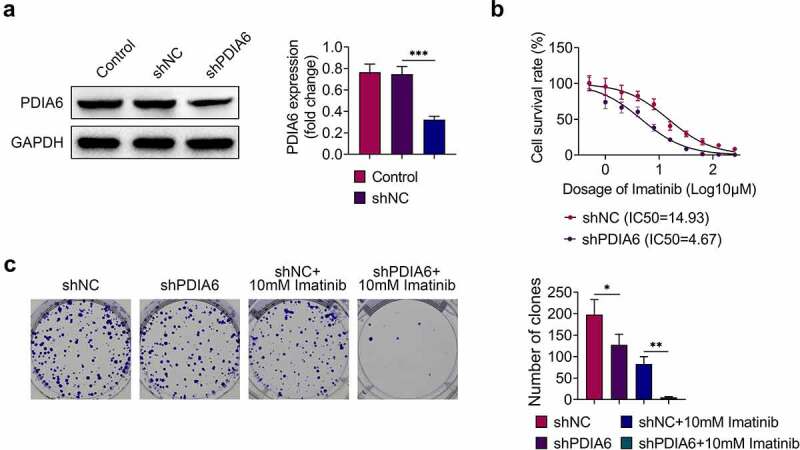
Knockdown of PDIA6 suppressed cell proliferation of imatinib-resistant renal cell carcinoma (a)Transfection with shPDIA6 reduced protein expression of PDIA6 in A498/R. (b) Knockdown of PDIA6 decreased IC50 of A498/R compared to the control (4.67 μM vs. 14.93 μM). (c) Knockdown of PDIA6 suppressed cell proliferation of A498/R, further reduced the cell proliferation of A498/R under imatinib condition. *, **, ***, *p* < 0.05, *p* < 0.01, *p* < 0.001

A498/R was transfected with shPDIA6 to investigate the effects of PDIA6 on imatinib-resistance of renal cell carcinoma. The transfection efficiency was confirmed in Figure 2(a). Knockdown of PDIA6 decreased IC50 of A498/R compared with the control (4.67 μM vs. 14.93 μM) (Figure 2(b)). Moreover, cell proliferation of A498/R cells was repressed by transfection with shPDIA6 (Figure 2(c)), and knockdown of PDIA6 further reduced the cell proliferation of A498/R upon imatinib treatment (Figure 2(c)). These results demonstrated that PDIA6 contributed to the cell proliferation of imatinib-resistant renal cell carcinoma.

### Knockdown of PDIA6 promoted cell apoptosis of imatinib-resistant renal cell carcinoma

**Figure 3.**
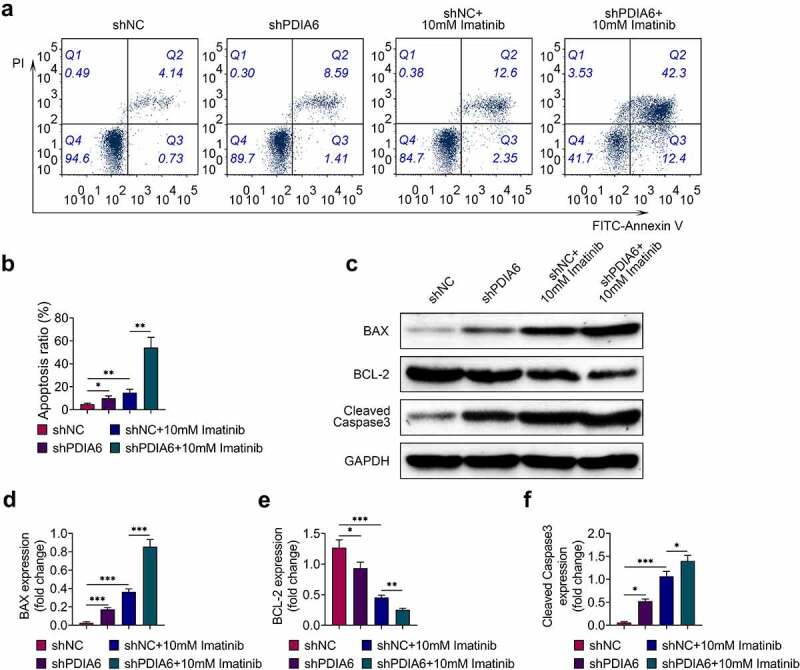
Knockdown of PDIA6 promoted cell apoptosis of imatinib-resistant renal cell carcinoma (a) Knockdown of PDIA6 promoted cell apoptosis of A498/R, and further promoted the cell apoptosis of A498/R under imatinib condition. (b) Knockdown of PDIA6 promoted cell apoptosis ratio of A498/R, and further promoted the cell apoptosis ratio of A498/R under imatinib condition. (c) Knockdown of PDIA6 reduced Bcl-2, enhanced Bax and cleaved caspase-3 in A498/R, and further reduced Bcl-2, enhanced Bax and cleaved caspase-3 in A498/R under imatinib condition. (d) The fold change of Bax in A498/R with transfection of shNC or sh PDIA6 post or without imatinib condition. (e) The fold change of Bcl-2 in A498/R with transfection of shNC or sh PDIA6 post or without imatinib condition. (f) The fold change of cleaved caspase-3 in A498/R with transfection of shNC or sh PDIA6 post or without imatinib condition. *, **, ***, *p* < 0.05, *p* < 0.01, *p* < 0.001

Cell apoptosis of A498/R was promoted by silencing of PDIA6 (Figure 3(a,b)). Imatinib condition aggravated the promotive effect of PDIA6 silencing on cell apoptosis of A498/R (Figure 3(a,b)). Protein expression of Bcl-2 was reduced, while Bax and cleaved caspase-3 were enhanced in A498/R by interference of PDIA6 (Figure 3(c-f)). Interference of PDIA6 further decreased the expression of Bcl-2, increased Bax and cleaved caspase-3 in A498/R post incubation with imatinib (Figure 3(c-f)). These results indicated that PDIA6 suppressed the cell apoptosis of imatinib-resistant renal cell carcinoma.

### Knockdown of PDIA6 suppressed DNA damage repair of imatinib-resistant renal cell carcinoma

**Figure 4.**
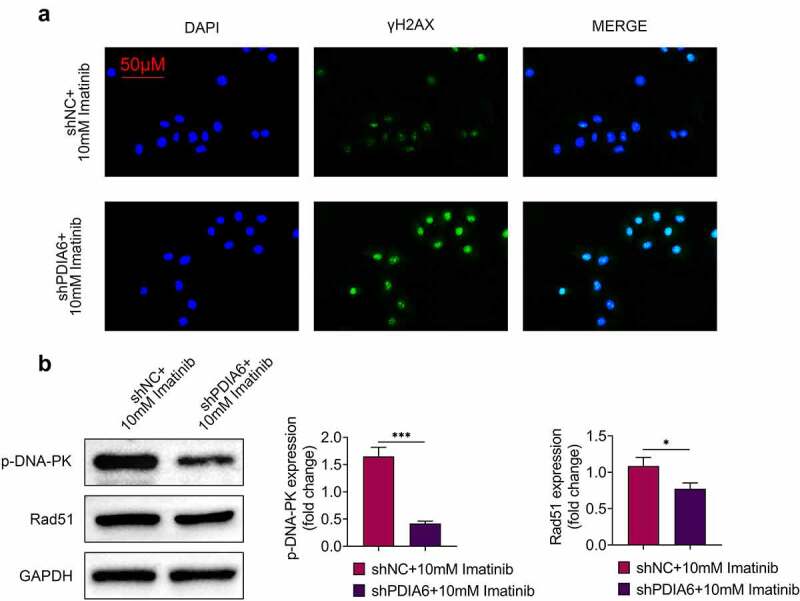
Knockdown of PDIA6 suppressed DNA damage repair of imatinib-resistant renal cell carcinoma (a) Knockdown of PDIA6 increased immunofluorescence of γH2AX in A498/R under imatinib condition. (b) Knockdown of PDIA6 down-regulated protein expression of p-DNA-PK and Rad51 in A498/R under imatinib condition. *, ***, *p* < 0.05, *p* < 0.001

Immunofluorescence of γH2AX was increased in A498/R under imatinib condition by silencing of PDIA6 (Figure 4(a)). Knockdown of PDIA6 down-regulated protein expression of p-DNA-PK and Rad51 in A498/R post incubation with imatinib (Figure 4(b)), revealing that PDIA6 contributed to the DNA damage repair of imatinib-resistant renal cell carcinoma.

Knockdown of PDIA6 suppressed activation of Wnt3a-FZD1 pathway in imatinib-resistant renal cell carcinoma

**Figure 5.**
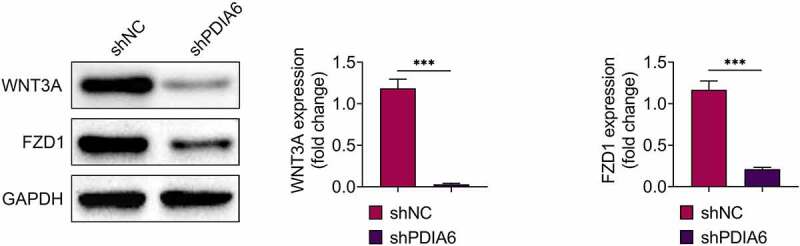
Knockdown of PDIA6 suppressed activation of Wnt3a-FZD1 pathway in imatinib-resistant renal cell carcinoma Knockdown of PDIA6 down-regulated protein expression of Wnt3a and FZD1 in A498/R. ***, *p* < 0.001

Protein expression levels of Wnt3a and FZD1 were down-regulated in A498/R (Figure 5), suggesting that PDIA6 contributed to the activation of Wnt3a-FZD1 pathway in imatinib-resistant renal cell carcinoma.

Over-expression of FZD1 attenuated the suppressive effect of PDIA6 silence on imatinib-resistant renal cell carcinoma

**Figure 6.**
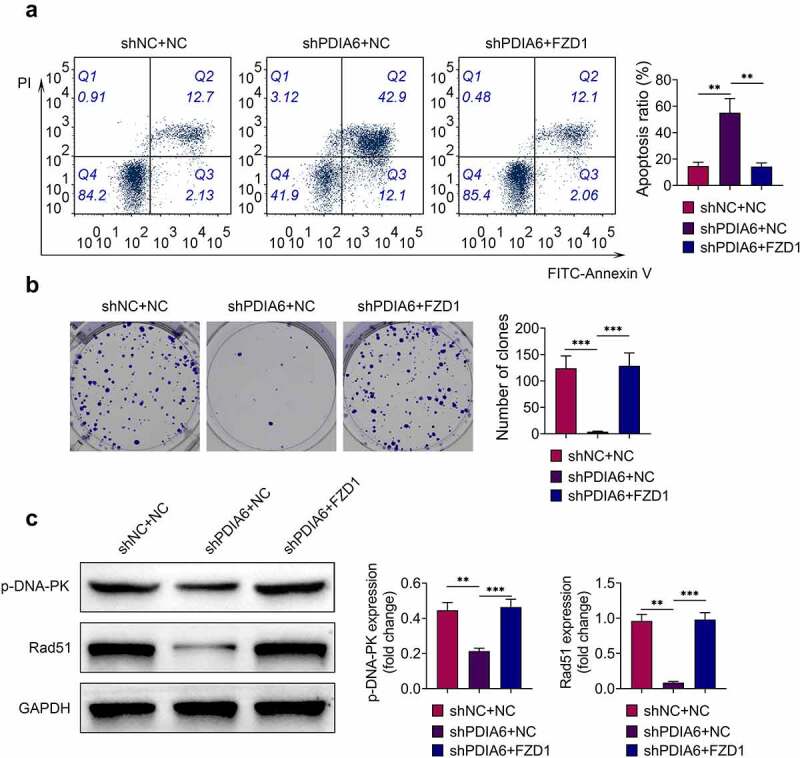
Over-expression of FZD1 attenuated the suppressive effect of PDIA6 silencing on imatinib-resistant renal cell carcinoma (a) Over-expression of FZD1 attenuated PDIA6 silencing-induced increase of cell apoptosis in A498/R. (b) Over-expression of FZD1 attenuated PDIA6 silencing-induced decrease of cell proliferation in A498/R. (c) Over-expression of FZD1 attenuated PDIA6 silencing-induced decrease of p-DNA-PK and Rad51 in A498/R. **, ***, *p* < 0.01, *p* < 0.001

To investigate the role of PDIA6/Wnt3a/FZD1 in imatinib-resistance of renal cell carcinoma, A498/R were cotransfected with shPDIA6 and pcDNA-FZD1. The increased cell apoptosis of A498/R by knockdown of PDIA6 was repressed by over-expression of FZD1 (Figure 6(a)). Over-expression of FZD1 reversed the suppressive effect of PDIA6 interference on cell proliferation of A498/R (Figure 6(b)). The down-regulated p-DNA-PK and Rad51 in A498/R with shPDIA6 transfection was up-regulated by the over-expression of FZD1 (Figure 6(c)), indicating that PDIA6 contributed to the imatinib-resistance of renal cell carcinoma through activation of Wnt3a-FZD1 pathway.

## Discussion

Protein disulfide isomerases are involved in the endoplasmic reticulum proteostasis and stress, thus contributing to the tumorigenesis [18]. Protein disulfide isomerases are also regarded as therapeutic targets for monoclonal antibodies during the immune-mediated tumor destruction [19]. Inhibition of protein disulfide isomerases has been used for cancer treatment [18]. Moreover, protein disulfide isomerases are also involved in the contribution of cancer cells to chemoresistance [20]. For example, PDIA5 modulated resistance of leukemia cells to imatinib treatment [21]. Since PDIA6 was found to be an oncogene and contribute to the chemoresistance of cancer cells, the effect of PDIA6 on imatinib-resistant renal cell carcinoma was investigated in this study.

PDIA6 was found to be up-regulated in imatinib-resistant renal cell carcinoma tissues and cells. Functional assays showed that knockdown of PDIA6 reduced IC50 of A498/R to imatinib and sensitized A498/R to imatinib through suppression of cell proliferation and promoting of cell apoptosis. Moreover, increased expression of anti-apoptotic proteins (Bcl-2, Bcl-xL) and decreased pro-apoptotic protein (Bax and Bad) have been reported to be involved in the resistance of clear-cell renal cell carcinoma cells to imatinib [15]. Knockdown of PDIA6 promoted cell apoptosis of cisplatin-resistant lung adenocarcinoma cells [13]. Our results showed that knockdown of PDIA6 decreased Bcl-2 expression, increased Bax, and cleaved caspase-3 to reduce imatinib-resistance of renal cell carcinoma cells. Previous study has shown that PDIA6 regulated c-Jun and ERK pathways to modulate caspase 3-mediated apoptosis and intrinsic mitochondrial apoptosis, thus participating in the sensitivity to cisplatin [22]. The role of PDIA6 in c-Jun and ERK pathways in imatinib-resistance of renal cell carcinoma should be investigated in further research.

Imatinib has been regarded as an apoptosis-inducing drug, and imatinib-resistant cells showed enhanced genomic instability through promoting of DNA damage repair [23]. γ-H2AX was down-regulated in imatinib-resistant chronic myeloid leukemia cells [24]. Here, the immunofluorescence of γH2AX in A498/R was upregulated by the knockdown of PDIA6. DNA damage repair biomarkers, p-DNA-PK and Rad51, were reduced by knockdown of PDIA6, suggesting that PDIA6 promoted DNA damage repair to enhance the resistance of renal cell carcinoma to imatinib.

PDIA6 has been shown to be essential for the folding of Wnt3a, and Wnt3a is a major ligand for the FZD receptor during the activation of Wnt pathway [25]. PDIA6 has been shown to promote the activation of Wnt/β-catenin pathway to contribute to the proliferation of HeLa cells [26]. Knockdown of PDIA6 reduced protein expression of Wnt3a and β-catenin in gastric cancer cells, and activation of Wnt pathway through incubation with lithium chloride attenuated PDIA6 silencing-induced increase in chemosensitivity [14]. Moreover, apolipoprotein C1 promoted Wnt3a to stimulate progression of renal cell carcinoma [27]. FZD1, upregulated in sunitinib-resistant clear cell renal cell carcinoma cells, was associated with the resistance of sunitinib [28]. Protein expression levels of Wnt3a and FZD1 in A498/R were reduced by PDIA6 knockdown, and over-expression of FZD1 attenuated the promotive effect of PDIA6 interference on cell apoptosis of A498/R, as well as the suppressive effect of PDIA6 interference on the cell proliferation. Inhibition of Wnt pathway impaired DNA damage repair and promoted radiosensitivity of radioresistant esophageal cancer cell [29]. Silencing of PDIA6-induced decreases in p-DNA-PK and Rad51 was restored by over-expression of FZD1, suggesting that PDIA6 might contribute to the resistance of renal cell carcinoma to imatinib through activation of Wnt3a/FZD1 pathway.

## Conclusion

In conclusion, knockdown of PDIA6 suppressed cell proliferation and DNA damage repair of imatinib-resistant renal cell carcinoma cell and promoted the cell apoptosis through down-regulation of Wnt3a and FZD1. These results provided a novel mechanism involved in PDIA6-mediated chemoresistance of renal cell carcinoma and highlighted the significance of PDIA6 inhibition as a potential therapeutic strategy for imatinib-resistant renal cell carcinoma.

## Highlight

- PDIA6 contributed to proliferation of imatinib-resistant renal cell carcinoma
- PDIA6 contributed to apoptosis of imatinib-resistant renal cell carcinoma
- PDIA6 contributed to DNA damage repair of imatinib-resistant renal cell carcinoma
- PDIA6 contributed to activation of Wnt3a-FZD1 pathway
